# Epidemiology of burn patients admitted in the Netherlands: a nationwide registry study investigating incidence rates and hospital admission from 2014 to 2018

**DOI:** 10.1007/s00068-021-01777-y

**Published:** 2021-08-31

**Authors:** Daan T. Van Yperen, Esther M. M. Van Lieshout, Michael H. J. Verhofstad, Cornelis H. Van der Vlies

**Affiliations:** 1 Trauma Research Unit, Department of Surgery, Erasmus MC, University Medical Center Rotterdam, P.O. Box 2040, 3000 CA Rotterdam, The Netherlands; 2 Burn Center Maasstad Hospital, Rotterdam, The Netherlands

**Keywords:** Burns, Epidemiology, Non-burn center, Burn center

## Abstract

**Purpose:**

The aim of this study was to gain insight into the epidemiology of burn patients admitted to a hospital without a burn center or referred to a burn center.

**Methods:**

This retrospective, nationwide, cohort study included patients with burns or inhalation trauma, admitted between 2014 and 2018, from a national trauma registry. The primary outcome measure was admission to a hospital with or without a burn center. Secondary outcome measures were patient and injury characteristics, Intensive Care Unit (ICU) admission and length of stay, and hospital length of stay (HLOS).

**Results:**

Of the 5524 included patients, 2787 (50.4%) were treated at a non-burn center, 1745 (31.6%) were subsequently transferred to a burn center, and 992 (18.0%) were primarily presented and treated at a burn center. The annual number of patients decreased from 1199 to 1055 (− 12.4%). At all admission locations, a clear incidence peak was observed in children ≤ 4 years and in patients of ≥ 80 years. The number of ICU admissions for the entire population increased from 201 to 233 (33.0%). The mean HLOS for the entire population was 8 (SD 14) days per patient. This number remained stable over the years in all groups.

**Conclusion:**

Half of all burn patients were admitted in a non-burn center and the other half in a burn center. The number and incidence rate of patients admitted with burns or inhalation trauma decreased over time. An increased incidence rate was found in children and elderly. The number of patients admitted to the ICU increased, whereas mean hospital length of stay remained stable.

**Supplementary Information:**

The online version contains supplementary material available at 10.1007/s00068-021-01777-y.

## Introduction

Since the late 1980’s, three designated burn centers have been established in the Netherlands to provide care for the severely burned patient. Together with specialized trauma centers and non-burn centers, they form a well-organized network of burn care. Depending on the injury severity, a burn patient benefits from treatment at a specialized burn center. For other patients, treatment at a non-burn center is sufficient. To provide optimal care for all burn patients, the Emergency Management of Severe Burns (EMSB) referral criteria have been implemented in the Netherlands for several years [1]. These criteria have been designed as a support tool for clinicians to decide whether a patient should be referred to a specialized burn center. Adherence to the EMSB referral criteria for patients presented to a non-burn center was 70.0% [2].

Since 2009, extensive injury and outcome registration for patients admitted to a Dutch burn center has been captured in the Dutch Burn Repository (DBR R3). This repository shows that the number of admissions in the Dutch burns centers has been increasing over the past decade, from 747 patients in 2014 up to approximately 915 in 2019 [3, 4]. Unfortunately, this database only collects data of patients being treated at a specialized burn center. For the patients treated at a hospital without a specialized burn center, no burn-specific registry is available regarding the number of patients, injuries, treatment, and outcome. However, all burn patients admitted to either a burn center or a non-burn center are registered in a nationwide trauma registry. This registry collects basic data on injury location and severity as well as on generic outcomes such as intensive care and hospital admission and mortality.

Although a solid burn care network has been established in the Netherlands already, it remains important to optimize the organization of burn care and treatment quality. Currently, data about the nationwide demographics of burn patients, and in particular those treated at a non-burn center, are not available. The study was not hypothesis driven, but was aimed to answer the research question about the incidence, case load, and basic health care use of burn patients admitted to hospitals with or without a specialized burn center. Data of this study were expected to provide insight into the number of admitted and referred burn patients in the Netherlands and, depending of the study results, further evaluation of burn center triage could or could not be relevant.

## Materials and methods

### Study design and setting

This was a retrospective, nationwide cohort study. Potential participants were selected from the Dutch National Trauma Registry (NTR). This registry collects data of all trauma patients admitted or transferred to a hospital in the Netherlands within 48 h after the accident, regarding acute trauma care, type of injury, injury severity, and mortality. A detailed injury description is not included in this database. This study was exempted by the Medical Research Ethics Committee Erasmus MC (Rotterdam, The Netherlands; registration number MEC-2019-0144), and the need for informed consent was waived.

### Participants

All patients with burns or inhalation trauma admitted to a Dutch hospital between January 1, 2014 and December 31, 2018, and registered in the NTR, were eligible for inclusion. Participants were identified by searching the NTR for patients with a registered Abbreviated Injury Scale (AIS) for burn injury and inhalation trauma (Supplemental Table S1) [5]. The population was split based on the type of injury; (1) patients with burns without inhalation trauma (burns), (2) patients with inhalation trauma without burns (inhalation), and (3) patients with both burns and inhalation trauma (combined). Both suspected and confirmed injuries to the respiratory tract (i.e., with or without diagnostic test such as bronchoscopy) are encoded as inhalation trauma. Patients transferred from or to a foreign or unknown hospital were excluded as data for those hospitals were not available. Patients transferred < 48 h from a burn center to a non-burn center were excluded, as that transfer was not the research question. Patients were divided into three groups: (1) patients primarily presented and admitted to a hospital without a burn center (non-burn center group), (2) patients primarily presented to a non-burn center and subsequently transferred to a specialized burn center (transferred group), and (3) patients primarily presented and admitted to a burn center (burn center group).

### Outcome measures and data collection

The primary outcome measure in this study was admission to a hospital with or without a specialized burn center. Hospitals were categorized into two groups: hospitals without a burn center (non-burn center) and with a burn center (burn center). Based on this information, the location of hospital admission was determined for each patient (presentation to and treatment in a non-burn center, primary presentation to a non-burn center and immediate (i.e. < 48 h) transfer to a burn center, and presentation and treatment in a burn center).

Secondary outcome measures were patient and injury characteristics, the presence of additional injuries, and mortality. Patient and injury characteristics included age, gender, type of injury based on AIS coding (burns, inhalation trauma, and combined), year of trauma, and Injury Severity Score (ISS). Inhalation trauma also included patients who were only suspected to have inhalation injury and patients with carbon monoxide poisoning. An ISS ≥ 16 was considered as polytrauma. Additional injuries were defined as a registered AIS code of ≥ 1 (presence of any additional injury) or ≥ 3 (presence of severe additional injury) for each of the nine anatomic regions other than the burn or inhalation trauma. Treatment outcome included intensive care unit (ICU) admission and duration, hospital length of stay (HLOS), and mortality < 30 days after admission.

### Statistical analysis

Data were analyzed using the Statistical Package for the Social Sciences (SPSS) version 25.0 (SPSS, Chicago, Ill., USA). Normality of continuous data was tested with the Shapiro–Wilk test. Missing values were not imputed. Data were reported following the ‘Strengthening the Reporting of Observational studies in Epidemiology’ (STROBE) guidelines [6].

For continuous data, median and quartiles (non-normal distribution) or mean and standard deviation (SD; normal distribution) were reported. For categorical data, number and frequencies are reported. No statistical comparison was made between the groups.

Descriptive statistics were used to report the outcome measures. Data are shown for the entire population, as well as for the groups by admission location (non-burn center, transferred, and burn center), year of admission (2014–2018), age (per 5-year age group), type of injury (burns, inhalation trauma, or combined), and gender. Incidence rates per 100,000 persons years (py) were calculated from data about the Dutch mid-year standard population obtained from Statistics Netherlands [7]. The cumulative ICLOS and HLOS were calculated by multiplying the mean ICLOS and HLOS per patient with the number of patients in that group.

### Patient and public involvement

The need for this study emerged from meetings of the Association of Dutch Burn Centers as well as the Dutch Burns Foundation, representing both health-care professionals and patients. Input into the design was given during presentation for the Scientific Board of the Dutch Burns Foundation. Patients were not directly involved in the subsequent writing of the study protocol, nor were they directly involved in the conduct of the study.

## Results

### Patient selection

**Fig. 1:**
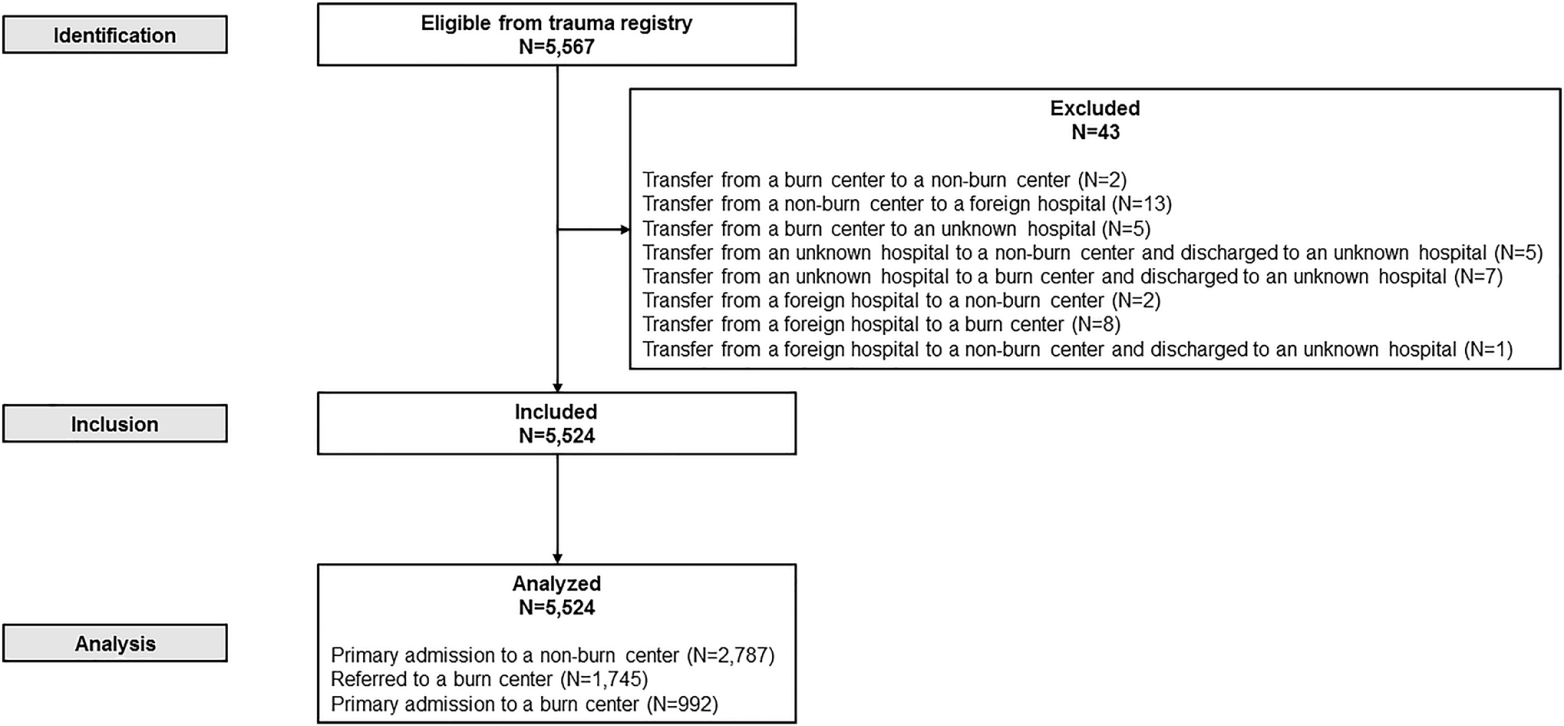
Study flowchart

From 2014 to 2018, 5567 patients with burn wounds were registered in the NTR. For the analysis, 43 patients were excluded, leaving 5524 patients included in the analysis (Fig. 1). A total of 4532 (81.4%) patients primarily presented to a non-burn center, of whom 1745 were subsequently transferred to a burn center. The remaining 922 patients were presented to a specialized burn center. Of all patients, 2787 (50.4%) received their primary treatment in a non-burn center and 2737 (49.5%) in a burn center.

### Patient characteristics, injury details, and mortality

Burn wounds were the most common injuries (*N* = 4316; 78.1%), followed by inhalation trauma (*N* = 810; 14.7%), and combined injury (*N* = 398; 7.2%; Table 1). The proportion of patients with inhalation trauma was higher in patients treated at a non-burn center (*N* = 750; 26.9%) than in patients transferred to a burn center (*N* = 9; 1%) or treated at a burn center (*N* = 51; 5%). Most patients had burns with a minor injury severity; of all patients, 3458 (73.4%) had an AIS of 1. This was the case in 1618 (79.4%) patients in the non-burn center group, 1092 (62.9%) patients in the transferred group, and 748 (79.5%) in the burn center group. The overall mortality was 3.3% (*N* = 182), which was about the same for groups.

**Table 1:** Patient characteristics, injury details, and mortality for patients admitted to a non-burn center, or transferred or admitted to a burn center

	Non-burn center (*N* = 2787)	Transferred (*N* = 1745)	Burn center (*N* = 992)	Total (*N* = 5524)
Patient characteristics				
Age (years)	34 (25)^a^	27 (25)^a^	31 (25)	32 (25)^b^
Children < 5 years	459 (16.5%)^a^	580 (33.3%)^a^	254 (25.6%)	1293 (23.4%)^b^
Elderly ≥ 70 years	257 (9.2%)^b^	121 (6.9%)^b^	81 (8.2%)	459 (8.3%)^a^
Male	1870 (67.1%)^b^	1125 (64.5%)^b^	649 (65.4%)	3644 (66.0%)^a^
Injury characteristics				
Injury				
Burns	1877 (67.3%)	1576 (90.3%)	863 (87.0%)	4316 (78.1%)
Inhalation	750 (26.9%)	9 (0.5%)	51 (5.1%)	810 (14.7%)
Combined	160 (5.7%)	160 (9.2%)	78 (7.9%)	398 (7.2%)
Burn wound	2037 (73.1%)	1736 (99.5%)	941 (94.9%)	4714 (85.3%)
Maximum AIS				
1	1618 (79.4%)	1092 (62.9%	748 (79.5%)	3458 (73.4%)
2	283 (13.9%)	357 (20.6%	106 (11.3%)	746 (15.8%)
3	80 (3.9%)	191 (11.0%)	32 (3.4%)	303 (6.4%)
4	11 (0.5%)	32 (1.8%)	18 (1.9%)	61 (1.3%)
5	32 (1.6%)	62 (3.6%)	30 (3.2%)	124 (2.6%)
6	13 (0.6%)	2 (0.1%)	7 (0.7%)	22 (0.5%)
Inhalation trauma	910 (32.7%)	169 (9.7%)	129 (13.0%)	1208 (21.9%)
Maximum AIS				
1	0 (0.0%)	0 (0.0%)	0 (0.0%)	0 (0.0%)
2	686 (75.4%)	84 (49.7%)	95 (73.6%)	865 (71.6%)
3	131 (14.4%)	34 (20.1%)	14 (10.9%)	179 (14.8%)
4	60 (6.6%)	29 (17.2%)	12 (9.3%)	101 (8.4%)
5	30 (3.3%)	21 (12.4%)	7 (5.4%)	58 (4.8%)
6	3 (0.3%)	1 (0.6%)	1 (0.8%)	5 (0.4%)
Injury Severity Score	5 (9)	5 (7)	4 (9)	5 (8)
Admission and mortality				
ICU admission	390 (14.0%)^d^	297 (22.8%)^e^	217 (21.9%)^f^	1004 (18.2%)^c^
ICLOS per patient (days)	4 (7)	9 (13)	11 (15)	7 (12)
HLOS per patient (days)	4 (7)	13 (16)	13 (17)	8 (14)
Mortality	90 (3.2%)	53 (3.0%)	39 (3.9%)	182 (3.3%)
Mortality ≤ 30 days	86 (95.6%)	44 (83.0%)	33 (84.6%)	163 (89.6%)

Data are shown as mean (SD) or as *N* (%)

Data missing for ^a^1 patients, ^b^2 patient, ^c^51 patients, ^d^19 patients, ^e^24 patients, and ^f^8 patients

*AIS* Abbreviated Injury Scale, *ISS* Injury Severity Score, *ICU* intensive care unit, *ICLOS* intensive care unit length of stay, *HLOS* hospital length of stay, *SD* standard deviation

### Numbers and incidence rates

**Fig. 2:**
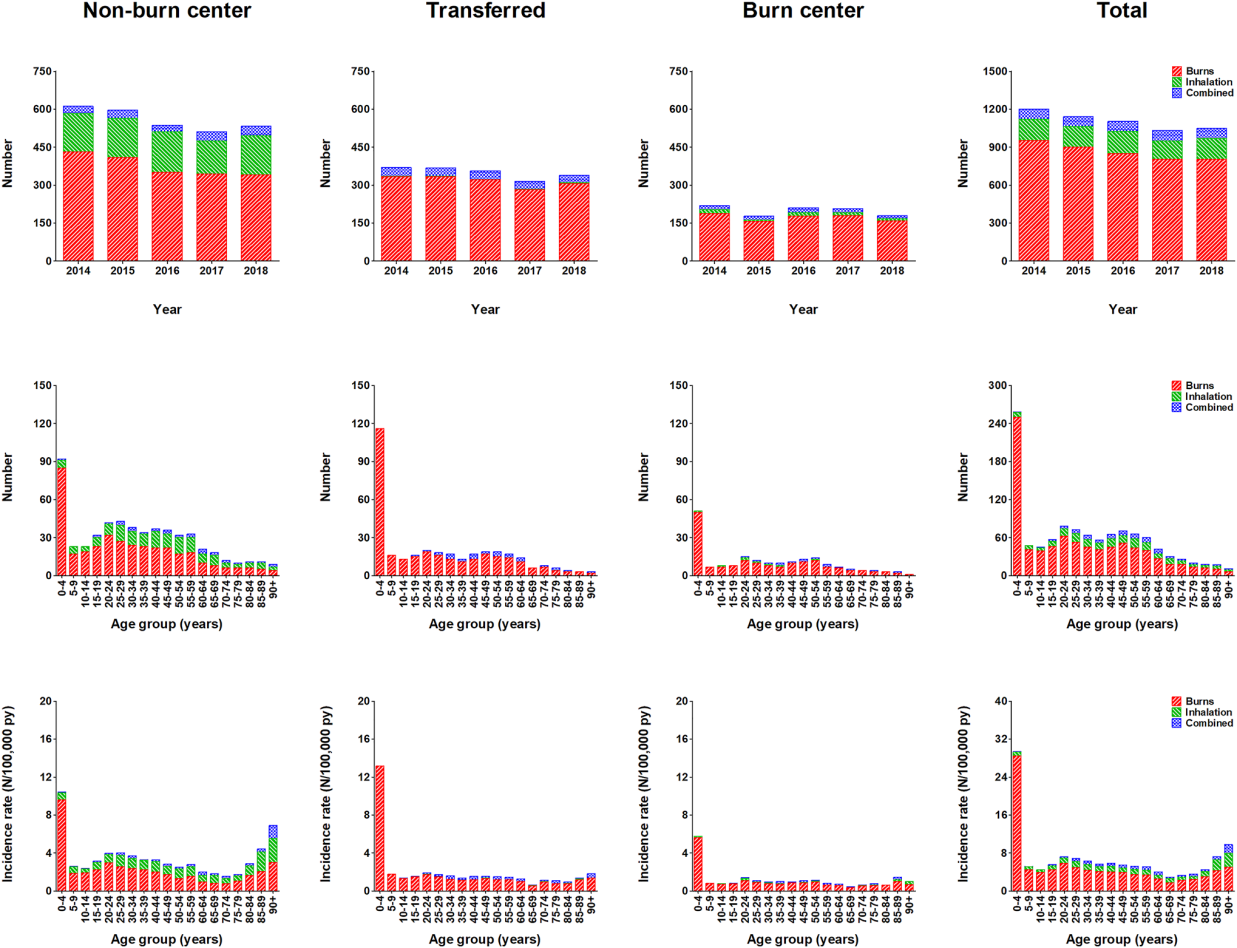
Numbers and incidence rates per year and age category, for each type of injury. For all admission locations and types of injuries, this figure shows the number of admissions per year, the overall number of admissions per age group, and the overall incidence rate per age group

The total number of patients admitted with burns decreased by 12.4%, from 1199 patients in 2014 to 1055 in 2018 (Fig. 2). This decrease was most pronounced in the burn center group (18.3%; from 219 to 179), followed by the non-burn center group (12.9%; 611–532), and the transferred group (8.1%; 369–339). In all groups, children ≤ 4 years accounted for the largest proportion of patients, with a mean of 259 (23.4%) per year. The overall incidence rate was 6.5/100,000 py (Table 2). A peak was observed in children ≤ 4 years (29.4/100,000 py), and to a lesser extent also in patients aged ≥ 80 (6.2/100,000 py) years. This was mainly seen in the non-burn center group.

**Table 2:** Incidence rate of intensive care unit admission and length of stay and hospital length of stay for patients admitted to a non-burn center or transferred or admitted to a burn center

	Age (year)	Non-burn center (*N* = 2787)				Transferred (*N* = 1745)				Burn center (*N* = 992)				Total (*N* = 5524)			
		Burns	Inhalation	Combined	Total	Burns	Inhalation	Combined	Total	Burns	Inhalation	Combined	Total	Burns	Inhalation	Combined	Total
Incidence rate (*N*/100.000 py)	≤ 4	9.6	0.7	0.1	10.3	13.2	0.0	0.0	13.2	5.7	0.1	0.0	5.8	28.4	0.8	0.1	29.4
	5–19	2.0	0.6	0.1	2.7	1.5	0.0	0.0	1.6	0.8	2.5	0.0	0.8	4.3	0.6	0.1	5.1
	20–64	1.9	1.0	0.2	3.1	1.3	0.0	0.3	1.6	0.8	0.1	0.1	1.0	4.1	1.1	0.6	5.7
	≥ 65	1.1	0.9	0.2	2.3	0.8	0.0	0.2	1.0	0.6	0.1	0.1	0.7	2.5	1.0	0.5	3.9
	≥ 80	2.0	1.6	0.5	4.0	1.0	0.1	0.2	1.2	0.8	0.1	0.1	1.0	3.7	1.7	0.7	6.2
	All	2.2	0.9	0.2	3.3	1.9	0.0	0.2	2.1	1.0	0.1	0.1	1.2	5.1	1.0	0.5	6.5
ICU (%)	≤ 4	34.4	50.0	6.8	6.4	0.0	0.0	6.4	6.0	0.0	0.0	5.9	5.6	29.7	50.0	6.4	34.4
	5–19	22.2	33.3	11.3	13.4	0.0	100.0	15.3	14.3	25.0	100.0	16.1	10.6	22.3	57.9	13.3	22.2
	20–64	24.6	29.6	15.2	21.5	100.0	90.1	33.5	18.7	41.2	90.8	29.3	14.9	26.4	68.8	22.7	24.6
	≥ 65	32.8	36.1	21.2	28.8	100.0	91.7	40.5	22.4	50.0	81.8	30.8	18.4	35.6	62.0	27.7	32.8
	≥ 80	33.9	29.4	22.0	37.8	100.0	100.0	50.0	31.0	75.0	66.7	38,9	22.1	38.5	51.9	30.2	33.9
	All	26.3	31.9	14.0	15.4	100.0	90.6	22.7	14.8	37.3	89.7	21,9	11.9	27.8	66.8	18.2	26.3
ICLOS (days/patient)	≤ 4	4	2	11	4	5	0	0	5	5	0	0	5	5	2	11	4
	5–19	3	7	5	5	5	0	12	6	2	2	12	3	4	6	9	5
	20–64	6	3	2	5	9	5	11	10	9	3	17	12	8	3	11	8
	≥ 65	3	4	3	4	8	9	7	7	12	8	6	9	8	5	6	6
	≥ 80	2	4	5	4	3	10	6	5	8	4	1	6	5	4	5	5
	All	5	4	3	4	8	7	11	9	8	4	16	11	7	4	11	7
Cumulative ICLOS (days)	≤ 4	12	5	4	21	27	0	0	27	13	0	0	13	51	5	4	61
	5–19	12	25	4	41	27	0	12	39	7	0	5	12	47	25	21	93
	20–64	99	89	16	227	229	6	261	512	140	7	208	359	468	102	485	1098
	≥ 65	10	38	9	56	53	7	32	91	45	6	10	61	107	51	50	208
	≥ 80	3	14	5	23	9	4	9	22	15	2	0	17	27	21	14	62
	All	134	156	33	323	336	13	305	654	204	13	223	440	674	183	560	1417
HLOS (days/patient)	≤ 4	2	2	10	2	8	0	0	8	7	2	0	7	6	2	10	6
	5–19	3	3	3	3	9	0	28	9	8	2	16	8	6	3	11	6
	20–64	4	3	2	4	14	8	25	17	13	3	34	16	9	3	19	9
	≥ 65	7	5	4	6	24	12	18	23	20	8	9	18	15	6	9	12
	≥ 80	9	6	4	7	26	13	19	24	16	10	2	14	15	7	8	12
	All	4	4	3	4	12	11	24	13	12	3	32	13	8	4	17	8
Cumulative HLOS (days)	≤ 4	205	14	8	227	886	0	0	886	353	2	0	355	1444	16	8	1468
	5–19	152	63	8	223	395	0	28	422	180	1	6	187	726	64	42	832
	20–64	746	315	56	1208	1921	10	654	2731	1121	19	473	1,681	3788	344	1183	5620
	≥ 65	236	148	30	413	611	10	84	705	333	13	20	365	1179	171	134	1483
	≥ 80	128	75	13	217	190	5	27	222	91	8	1	101	409	89	41	539
	All	1,339	540	101	1980	3812	20	766	4598	1986	36	498	2,520	7137	595	1366	9097

*Py* person years, *ICU* intensive care unit, *ICLOS* intensive care unit length of stay, *HLOS* hospital length of stay

For the entire population, inhalation trauma—isolated or as part of a combined injury—in particular was present among patients aged 25–59 years (Fig. 2). Isolated inhalation trauma was observed only in the non-burn center group and combined injury was mainly present in transferred patients, though to a very limited extent (*N* = 32; 9%). Remarkably, the incidence rate of isolated inhalation trauma and inhalation trauma in combination with burns showed a peak among patients ≥ 80 years treated at a non-burn center, with 1.6 and 0.5/100,000 py, respectively (Table 2). Additional information about the mean numbers and incidence rates per age category for males and females is presented in Supplemental Figure S2.

### Intensive care unit admission and length of stay

**Fig. 3:**
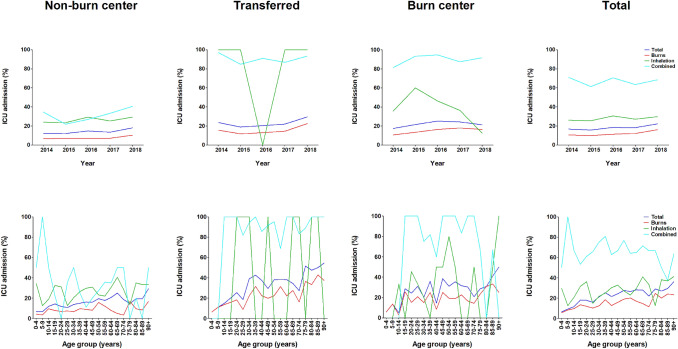
Intensive care unit admission per year, age category, and type of injury. For all admission locations and types of injuries, this figure shows the ICU admission rate per year and the average ICU admission rate per age group

On average, 201 (18.2%) patients are admitted to the ICU every year, in particular patients with combined injuries (66.8%) and inhalation injuries (27.8%) (Fig. 3).The number of ICU admissions increased by 33.0%, from 200 (16.7%) patients in 2014 to 233 (22.2%) patients in 2018. The highest number of ICU admissions was found in the transferred group (*N* = 397; 22.8%). ICU admission was gradually related to age in all groups.

The mean intensive care length of stay (ICLOS) remained stable over the years in all groups (Fig. 3). ICLOS was 7 (SD 12) days for the entire group (Table 1). This was 11 (SD 15) days for the burn center group, 9 (SD 13) for the transferred group, and 4 (SD 7) for the non-burn center group. Patients with combined injuries had the longest ICLOS: 10.7 days.

The cumulative ICLOS increased by 44.3%, from 1218 days in 2014 to 1747 in 2017. From then it decreased by 21.4% to 1381 days in 2018 (Supplemental Figure S3). Patients from the transferred group accounted for the greatest cumulative ICLOS, on average 654 days per year (Table 2).

### Hospital length of stay

**Fig. 4:**
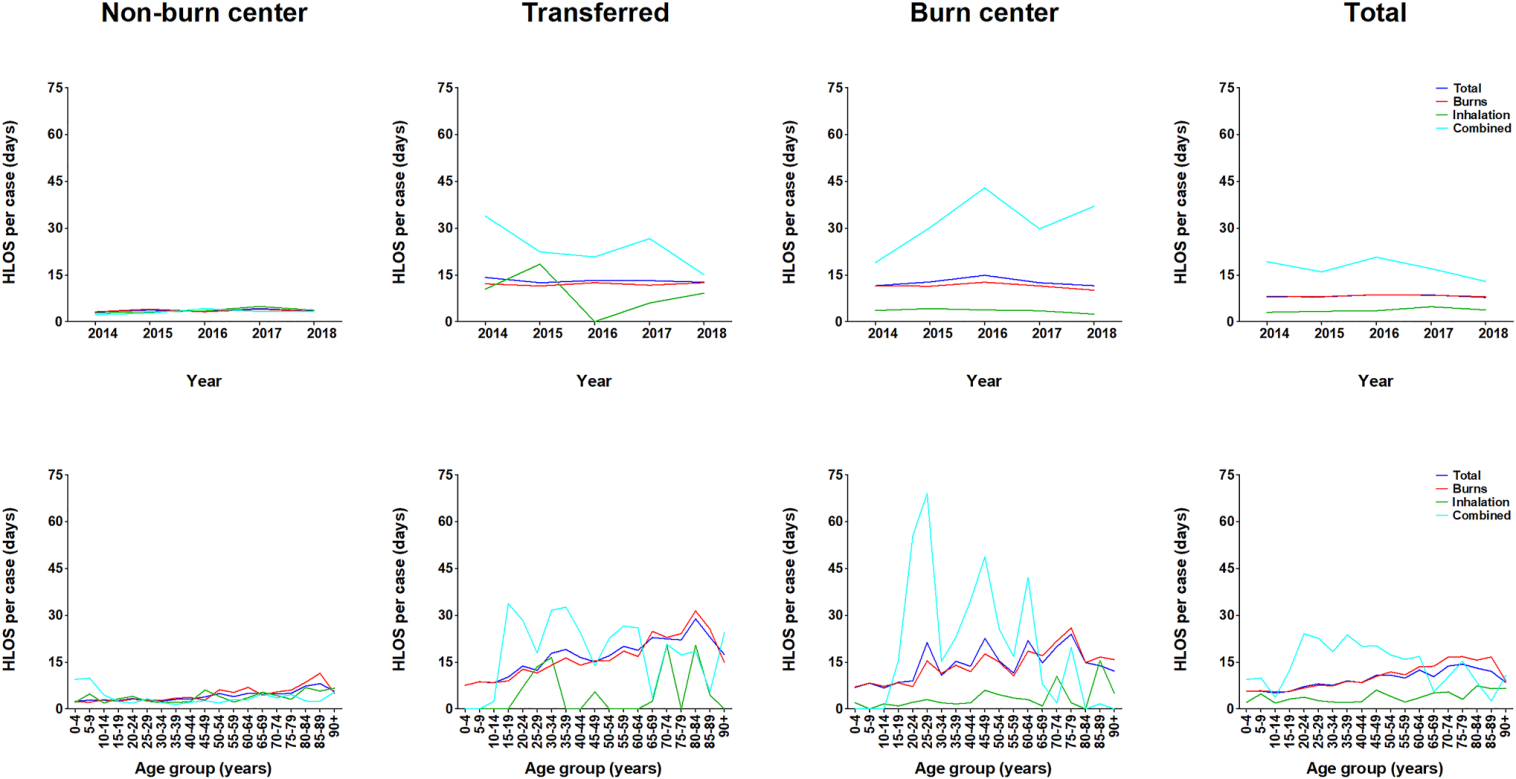
Hospital length of stay, per year, age category, and type of injury. For all admission locations and types of injuries, this figure shows the hospital length of stay per case per year and the average hospital length of stay per age group

The mean HLOS for the entire population was 8 (SD 14) days per patient (Table 1), and remained stable over the years in all groups (Fig. 4). The highest mean HLOS was found in the transferred group (13 days; SD 16) and the burn center group (13 days; SD 17), followed by the non-burn center group (4 days; SD 7). In all groups, except for the non-burn center group, the highest HLOS was seen in patients with combined injury (Fig. 4). HLOS gradually increased with age in all groups.

The cumulative HLOS decreased by 15.3%, from 9667 days in 2014 to 8187 in 2018 (Supplemental Figure S4). In all groups, burn wounds accounted for the largest proportion of the cumulative HLOS. HLOS was lowest in the non-burn center group, with a mean of 1980 days, followed by the burn center group with 2520 days, and the transferred group with 4599 days. In the entire population, patients aged ≤ 4 years accounted for the most hospital days, with a mean of 1468 days (Table 2).

## Discussion

The aim of this study was to gain insight into the epidemiology of burn patients admitted to a hospital without a specialized burn center or referred to a specialized burn center. This study showed that half of all burn patients were admitted to non-burn centers, and that the number and incidence rate of admitted burn patients has been decreasing. An increased incidence rate was found among children and elderly. Furthermore, the data showed that the number of ICU admissions increased whereas the hospital length of stay remained stable.

The overall number of patients treated annually for burn or inhalation injury decreased. This is in line with worldwide observations [8–12], which might indicate that the world is getting safer. Presumably, improved fire safety and changed human behavior play a role in this phenomenon. Another possibility is that burn injuries have become less severe, which is supported by Smolle et al., showing a decreasing burn injury severity on a worldwide scale [8].

The overall incidence rate found in this study was 6.5/100,000 py. The incidence rate of 8.5/100,000 reported by Dokter et al*.* in 2011 shows that there is an ongoing downward trend in the Netherlands [3]. However, a different strategy for patient selection was, which might influence these results. From 1985 to 2009, the incidence of burns in European countries varied between 2 and 29/100,000 py. Nordic countries, whom are similar to the Netherlands in many ways, show much higher incidence rates than those in our study. In Finland, Sweden, and Norway, incidence rates of 17.0, 15.5, and 12.4 per 100,000 inhabitants were found [13–15]. This might be explained by differences in health care system, climate, demography, and culture. Possibly, greater distances between hospitals in Nordic countries results in a lower threshold for admitting patients.

This study showed that the patients from the transferred group are more severely injured, indicating that the referral systems works. Nevertheless, 160 (3%) patients with both burns and inhalation trauma (combined injuries) were not transferred to a burn center, while this was to be expected according to the referral criteria. The reason why they could range from logistical reasons to the result of consulting the burn center. Due to the impossibility in the NTR database to code inhalation trauma (with the suspicion of inhalation injury), some of these patients have been registered as having inhalation injury, while in fact they only suffered inhalation trauma. This may have resulted in over-registration of inhalation injury.

An increased incidence rate was found among children and elderly, which corresponds with other studies [3, 10, 14, 17–21]. The higher incidence rate in children most likely highlights the inability of children to recognize dangers (e.g., hot liquids standing on a table). For the elderly, deterioration in cognitive and physical capabilities seems to increase their proneness to burns (e.g., forgetting they are burning a candle). The peak in incidence among elderly was mainly seen among those treated at a non-burn center, while this group should have been referred to a burn center according to the referral criteria. Whether deviating from the guidelines was done in consultation with a burn center was not registered. Adding this item would be a possible improvement of the NTR database.

ICU admissions increased by 30%, which is equal to Gigengack et al*.*, reporting an increase of at least 30% over the past 30 years in a Dutch burn center [22]. This study included many patients with small facial burns (77%). Treatment guidelines have emphasized the dangers of (possible) inhalation injury in patients with facial burns and encourage aggressive airway management. This has resulted in a lower threshold for observation at the ICU, resulting in increased ICU admissions.

Hospital length of stay in this study remained stable over time, which is in line with other studies [24–25]. However, on a worldwide scale most studies found a decline in HLOS [8, 9, 26, 27]. Compared with a mean of 10–33 days found in other countries, the mean HLOS in Dutch burn centers (13 days) is already at the lower end of the spectrum, and therefore a further decrease may not be expected [24–25, 28, 29].

### Strengths and limitations

A strength of this study is that it provides nationwide data about the epidemiology of burns, over multiple years, from burn center as well as non-burn centers.

A limitation of this study is that the NTR enters burn-specific items in less detail in comparison with the DBR R3. Due to different designs and privacy regulations, both registries cannot be merged into one. Investigating burn-specific questions for the non-burn centers may benefit from a nationwide burn-specific database. A strength of this study is that a rather homogenous group of trauma patients was taken as an example to show the potentials for improvements of the structure of the generic nationwide trauma registry: burn injury description, treatment, wound healing, and scar quality.

## Conclusion

The aim of this study was to gain insight into the epidemiology of burn patients admitted to a hospital without a specialized burn center or referred to a specialized burn center. Approximately, half of all patients received their treatment at a non-burn center. Over the past 5 years, the number and incidence rate of admitted patients with burns or inhalation trauma decreased by 12%, regardless of admission location. An increased incidence rate was found in children and elderly. The annual number of patients admitted to the ICU increased, whereas the hospital length of stay per patient remained stable during the study period.

The results of this study are important to optimize the organization of burn care and treatment quality. Furthermore, this article emphasizes that there is room for improvement in existing databases, to obtain complete and reliable data regarding burn patients.

## Supplementary Information

Below is the link to the electronic supplementary material.

Supplementary Figure S1. Numbers and incidence rate per age category for males and females. For all admission locations and gender, this figure shows the overall number of admissions and overall incidence rate per age group (TIF 792 KB)

Supplementary Figure S2. Intensive Care Unit length of stay per year, age category, and type of injury. For all admission locations and types of injuries, this figure shows the Intensive Care Unit length of stay per case per year and the average Intensive Care Unit length of stay per age group (TIF 571 KB)

Supplementary Figure S3. Cumulative Intensive Care Unit admission days per year, age category, and type of injury. For all admission locations and types of injuries, this figure shows the cumulative Intensive Care Unit length of stay per year and the average cumulative Intensive Care Unit length of stay per age group (TIF 1337 KB)

Supplementary Figure S4. Cumulative hospital admission days, per year, age category, and type of injury. For all admission locations and types of injuries, this figure shows the cumulative hospital length of stay per year and the average cumulative hospital length of stay per age group (TIF 464 KB)

Supplementary file5 (DOCX 36 KB)

Supplementary file6 (DOCX 35 KB)

## Data Availability

Data are not available for sharing because data ownership lies with a third party, and only aggregate data were available to the authors for analysis.
