# Supplementary figures and images for: Epidemiology of burn patients admitted in the Netherlands: a nationwide registry study investigating incidence rates and hospital admission from 2014 to 2018
Source: Eur J Trauma Emerg Surg. 2021 Aug 31;48(3):2029–38. doi: 10.1007/s00068-021-01777-y (PMC9192419; doi:10.1007/s00068-021-01777-y)

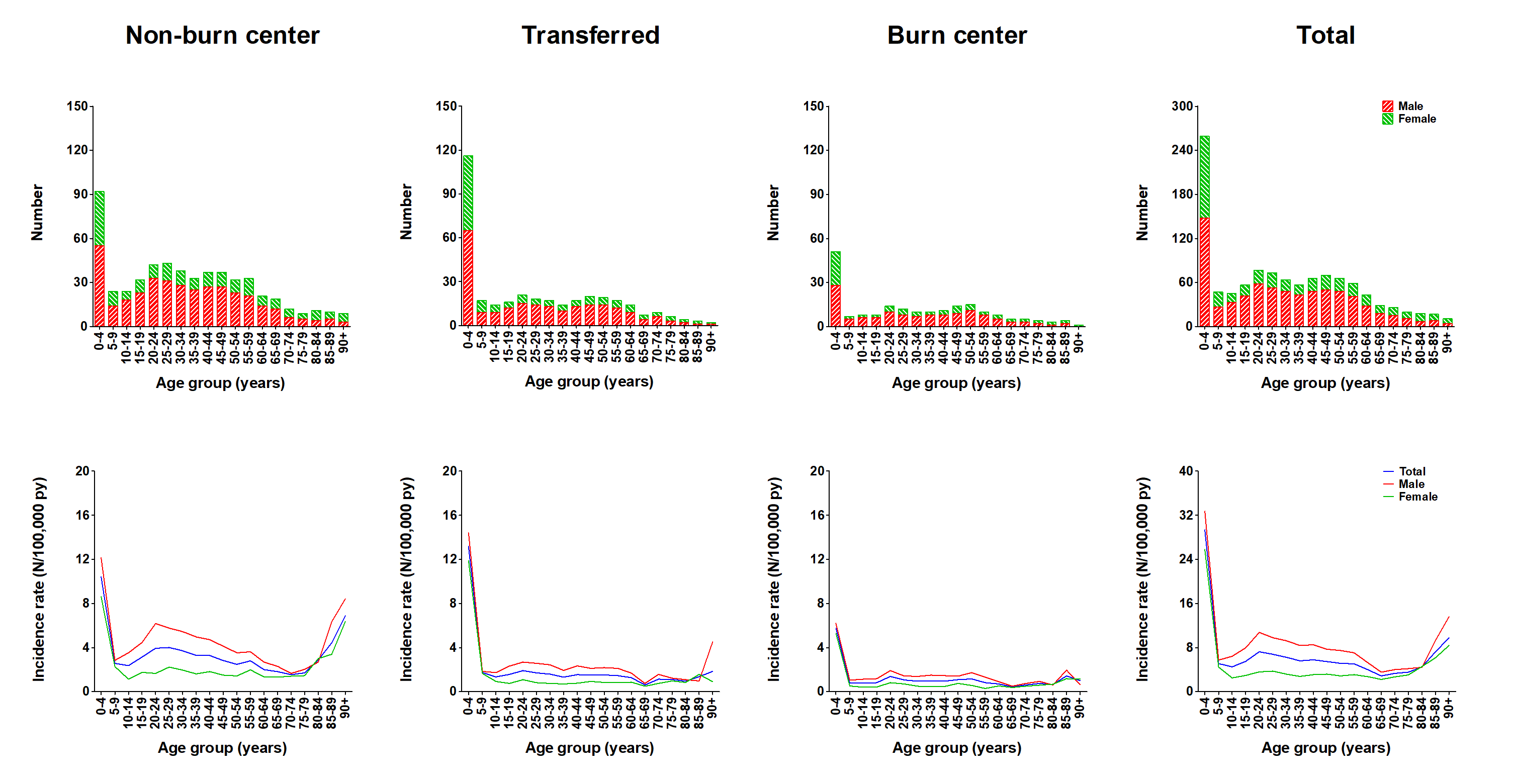

Supplement: Supplementary file 1 — Supplementary Figure S1. Numbers and incidence rate per age category for males and females. For all admission locations and gender, this figure shows the overall number of admissions and overall incidence rate per age group (TIF 792 KB) [file 68_2021_1777_MOESM1_ESM.tif]

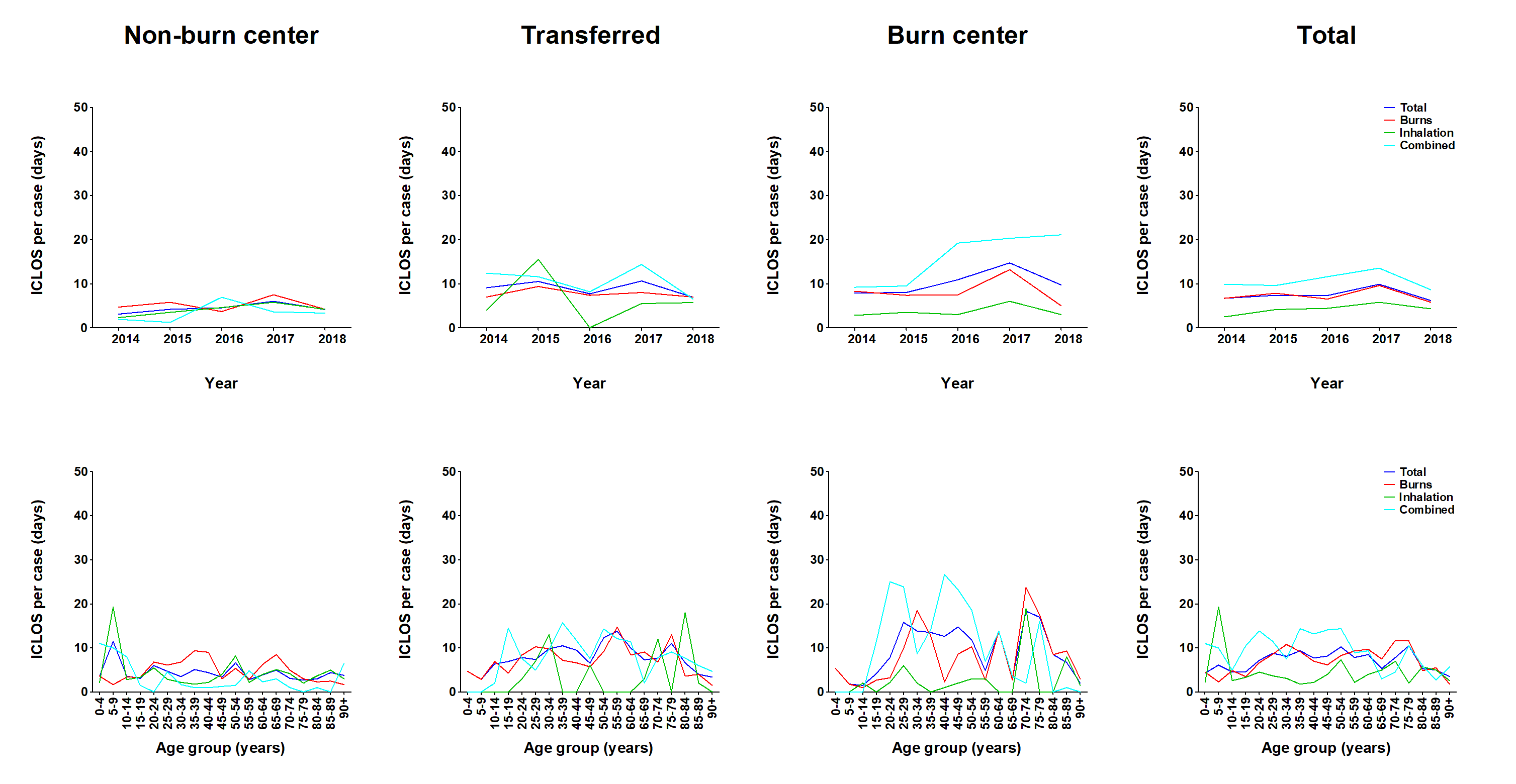

Supplement: Supplementary file 2 — Supplementary Figure S2. Intensive Care Unit length of stay per year, age category, and type of injury. For all admission locations and types of injuries, this figure shows the Intensive Care Unit length of stay per case per year and the average Intensive Care Unit length of stay per age group (TIF 571 KB) [file 68_2021_1777_MOESM2_ESM.tif]

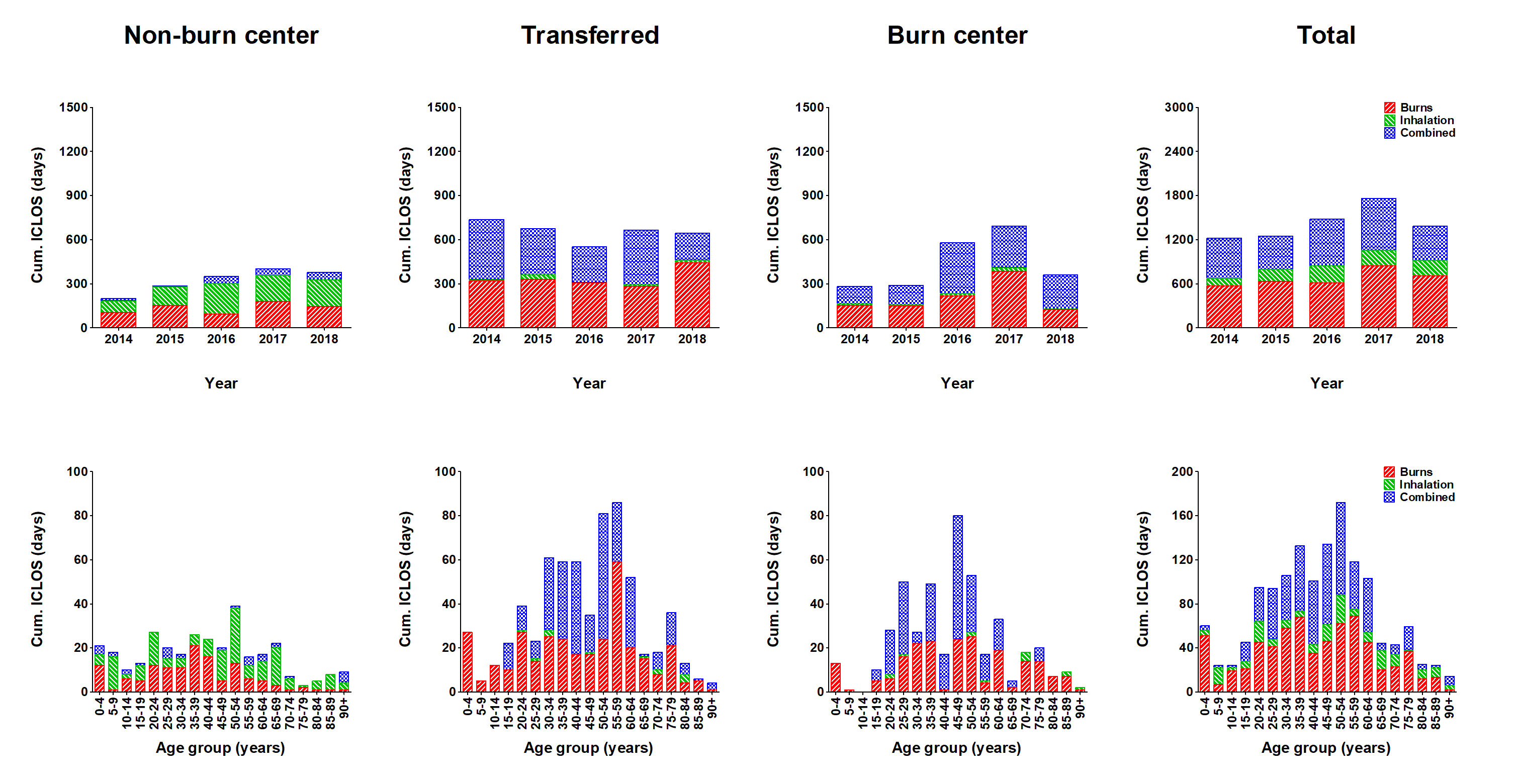

Supplement: Supplementary file 3 — Supplementary Figure S3. Cumulative Intensive Care Unit admission days per year, age category, and type of injury. For all admission locations and types of injuries, this figure shows the cumulative Intensive Care Unit length of stay per year and the average cumulative Intensive Care Unit length of stay per age group (TIF 1337 KB) [file 68_2021_1777_MOESM3_ESM.tif]
