# Supplementary material for: Epidemiology of burn patients admitted in the Netherlands: a nationwide registry study investigating incidence rates and hospital admission from 2014 to 2018
Source: Eur J Trauma Emerg Surg. 2021 Aug 31;48(3):2029–38. doi: 10.1007/s00068-021-01777-y (PMC9192419; doi:10.1007/s00068-021-01777-y)
Supplement: Supplementary file 5 — Supplementary file5 (DOCX 36 KB) [file 68_2021_1777_MOESM5_ESM.docx]

## Supplemental Table S1. Identification of patients in the National Trauma Registry with burns injury, inhalation injury or electrical injury according to the Abbreviated Injury Scale (AIS)

|  | **AIS-1998**  **(2014)** | **AIS-2005**  **(2015-2018)** | **Code description** | **% TBSA burned** |
| --- | --- | --- | --- | --- |
| **Burn injury** | 912000.1 | 912000.1 | NFS | NFS |
|  | 912002.1 | 912002.1 | 1^st^ degree; superficial; >1 year old | Any |
|  | 912003.1 | 912003.1 | ≤1 year old | ≤50% |
|  | 912004.2 | 912004.2 | ≥1 year old | >50% |
|  | 912006.1 | 912006.1 | 2^nd^ degree; partial thickness | <10% |
|  | 912007.1 | 912007.1 | 3^rd^ degree; full thickness | ≤100 cm^2^ [face ≤25 cm^2^] |
|  | 912008.2 | 912008.2 | 3^rd^ degree; full thickness | >100 cm^2^; <10% [face >25 cm^2^] |
|  | 912012.2 | 912012.2 | 2^nd^ or 3^rd^ degree; partial of full thickness | 10-19%^a^ |
|  | 912014.3 | 912014.3 | <5 years old | 10-19%^a^ |
|  | 912016.3 | X | Face/hand/genitalia involvement |  |
|  | 912018.3 | 912018.3 | 2^nd^ or 3^rd^ degree; partial or full thickness | 20-29^a^ |
|  | 912020.4 | 912020.4 | <5 years old | 20-29^a^ |
|  | 912022.4 | X | Face/hand/genitalia involvement |  |
|  | 912024.4 | 912024.4 | 2^nd^ or 3^rd^ degree; partial or full thickness | 30-39^a^ |
|  | 912026.5 | 912026.5 | <5 years old | 30-39^a^ |
|  | 912028.5 | X | Face/hand/genitalia involvement |  |
|  | 912030.5 | 912030.5 | 2^nd^ or 3^rd^ degree; partial or full thickness | 40-89% |
|  | 912032.6 | 912032.6 | 2^nd^ or 3^rd^ degree; partial or full thickness, including incineration | ≥90% |
|  | **AIS-1998**  **(2014)** | **AIS-2005**  **(2015-2018)** | **Code description** | |
| **Inhalation injury** | 919200.2 | 419200.2 | Inhalation injury NFS | |
|  | 919201.2 | 419201.2 | Absence of carbonaceous deposits, erythema, edema, bronchorrhea or obstruction | |
|  | 929102.3 | 419202.3 | Minor or patchy areas of erythema, carbonaceous deposits in proximal or distal bronchi | |
|  | 919204.4 | 419204.4 | Moderate degree of erythema, carbonaceous deposits, bronchorrhea with or without compromise of the bronchi | |
|  | 919206.5 | 419206.5 | Severe inflammation with friability, copious carbonaceous deposits, bronchorrhea, bronchial obstruction, hypoxemia | |
|  | 919208.6 | 419208.6 | Evidence of mucosal sloughing, necrosis, endoluminal obliteration | |
| **Electrical injury** | 919400.2 | 080000.2 | High voltage electrical injury | |
|  | 919402.3 | 080002.3 | With muscles necrosis | |
|  | 919404.5 | 080004.5 | With cardiac arrest documented by medical personnel | |

AIS, Abbreviated Injury Scale; TBSA, Total Body Surface Area; NFS, not further specified.

^a^ Any body region.
