# Supplementary material for: Epidemiology of burn patients admitted in the Netherlands: a nationwide registry study investigating incidence rates and hospital admission from 2014 to 2018
Source: Eur J Trauma Emerg Surg. 2021 Aug 31;48(3):2029–38. doi: 10.1007/s00068-021-01777-y (PMC9192419; doi:10.1007/s00068-021-01777-y)
Supplement: Supplementary file 6 — Supplementary file6 (DOCX 35 KB) [file 68_2021_1777_MOESM6_ESM.docx]

## Supplemental Table S2. Patient characteristics, injury details, and mortality for patients with burn wounds, inhalation trauma, or combined injuries

|  | **Burns**  **(N=4,316)** | **Inhalation**  **(N=810)** | **Combined**  **(N=398)** | **All**  **(N=5,524)** |
| --- | --- | --- | --- | --- |
| **Patient characteristics** |  |  |  |  |
| Age (years) | 28 (25)^a^ | 43 (23) | 48 (19) | 32 (25)^a^ |
| Children <5 years | 1,252 (29.0%)^a^ | 37 (4.6%) | 4 (1.0%) | 1,293 (23.4%)^a^ |
| Elderly ≥70 years | 297 (6.9%)^a^ | 105 (13.0%) | 57 (14.3%) | 459 (8.3%)^a^ |
| Male | 2,870 (66.5%)^a^ | 481 (59.4%) | 293 (73.6%) | 3,644 (66.0%)^a^ |
| **Injury characteristics** |  |  |  |  |
| Burn wound | 4,316 (100.0%) | 0 (0.0%) | 398 (100.0%) | 4,714 (85.3%) |
| Maximum AIS |  |  |  |  |
| 1 | 3,220 (74.6%) | 0 (0.0%) | 238 (59.8%) | 3,458 (73.4%) |
| 2 | 700 (16.2%) | 0 (0.0%) | 46 (11.6%) | 746 (15.8%) |
| 3 | 267 (6.2%) | 0 (0.0%) | 36 (9.0%) | 303 (6.4%) |
| 4 | 47 (1.1%) | 0 (0.0%) | 14 (3.5%) | 61 (1.3%) |
| 5 | 70 (1.6%) | 0 (0.0%) | 54 (13.6%) | 124 (2.6%) |
| 6 | 12 (0.3%) | 0 (0.0%) | 10 (2.5%) | 22 (0.5%) |
| Inhalation trauma | 0 (0.0%) | 810 (100.0%) | 398 (100.0%) | 1,208 (21.9%) |
| Maximum AIS |  |  |  |  |
| 1 | 0 (0.0%) | 0 (0.0%) | 0 (0.0%) | 0 (0.0%) |
| 2 | 0 (0.0%) | 617 (76.2%) | 248 (62.3%) | 865 (71.6%) |
| 3 | 0 (0.0%) | 107 (13.2%) | 72 (18.1%) | 179 (14.8%) |
| 4 | 0 (0.0%) | 52 (6.4%) | 49 (12.3%) | 101 (8.4%) |
| 5 | 0 (0.0%) | 32 (4.0%) | 26 (6.5%) | 58 (4.8%) |
| 6 | 0 (0.0%) | 2 (0.2%) | 3 (0.8%) | 5 (0.4%) |
| Injury Severity Score | 3.2 (6.2) | 7.7 (8.8) | 15.4 (15.3) | 4.8 (8.3) |
| **Admission and mortality** |  |  |  |  |
| Admission location |  |  |  |  |
| Non-Burn Center | 1,877 (43.5%) | 750 (92.6%) | 160 (40.2%) | 2,787 (50.5%) |
| Transfer to Burn Center | 1,576 (36.5%) | 9 (1.1%) | 160 (40.2%) | 1,745 (31.6%) |
| Burn Center | 863 (20.0%) | 51 (6.3%) | 78 (19.6%) | 992 (18.0%) |
| ICU admission | 513 (11.9%) | 225 (27.4%) | 266 (66.8%) | 1,004 (18.2%) |
| ICLOS (days) | 7 (11)^b^ | 4 (7)^c^ | 11 (15)^d^ | 7 (12)^a^ |
| HLOS (days) | 8 (12) | 4 (8) | 17 (28) | 8 (14) |
| Mortality | 83 (1.9%) | 36 (4.4%) | 63 (15.8%) | 182 (3.3%) |
| Mortality ≤30 days | 71 (85.5%) | 36 (100.0%) | 56 (88.9%) | 163 (89.6%) |

Data are shown as mean (SD) or as N (%).

Data missing for ^a^ 2 patients, ^b^ 51 patients, ^c^ 19 patients, ^d^ 24 patients.

AIS, Abbreviated Injury Scale; ISS, Injury Severity Score; ICU, Intensive Care Unit; ICLOS, Intensive Care Unit Length of Stay; HLOS, Hospital Length of Stay; SD, Standard Deviation.
